# What is Your Diagnosis?

**DOI:** 10.5812/iranjradiol.7607

**Published:** 2012-11-20

**Authors:** Pooyan Dehghani, Dorna Motevalli, Sanaz Asadian, Nasrin Saki

**Affiliations:** 1 Department of Cardiology, Shiraz University of Medical Sciences, Shiraz, Iran; 2 Students Research Center, Shiraz University of Medical Sciences, Shiraz, Iran; 3 Department of Radiology, Shiraz University of Medical Sciences, Shiraz, Iran; 4 Department of Dermatology, Skin Research Center , Shiraz University of Medical Sciences, Shiraz, Iran

**Keywords:** Dyspnea, Surgery, Cardiac

A 60-year-old man was referred with a 1-month history of angina and dyspnea to a cardiology clinic. In physical examination, there was a rough, low-pitched crescendo-decrescendo systolic murmur that was best heard at the second intercostal space at the right upper sternal border.

Echocardiography showed severe aortic stenosis and left ventricular hypertrophy. After angiography, he was planned for aortic valve replacement, but the patient still remained dyspneic after valve replacement. Posteroanterior chest radiography was performed.

1. What is the probable cause of the patient’s dyspnea?

2. What type of cardiac surgery has been performed for the patient?

**Figure:**
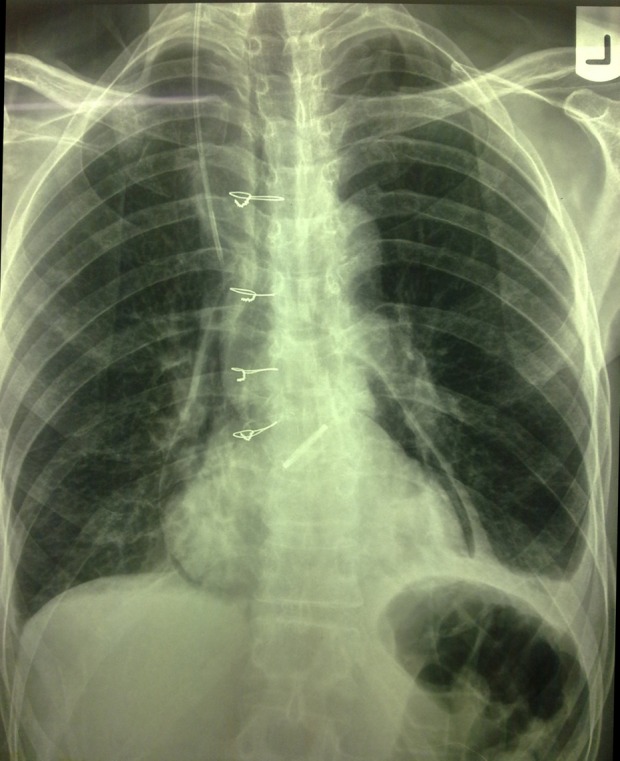


## Diagnosis: Post-Operation Pneumopericardium and Pneumomediastinum

Posteroanterior plain chest radiography shows a radiolucency surrounding the heart, separated from the lung with a thin white line of pericardium in favor of pneumopericardium. Some linear and curvilinear lucencies are seen within the mediastinal contours representing pneumomediastinum. Costophrenic angles are blunted bilaterally in favor of bilateral pleural effusion especially prominent on the left side.

Increased convexity of the left heart border and apex is noted associated with downward pointing of the apex indicative of left ventricular hypertrophy; however, the cardiothoracic ratio is in normal range. A device (prosthetic valve) is seen in the anatomic site of the aortic valve and the ascending aorta is enlarged indicative of post-stenotic dilatation. All these findings are in favor of severe aortic valve stenosis which has been corrected with aortic valve replacement. Note the increased diameter of the colon in the splenic flexure which has caused elevation of the left hemidiaphragm. This condition is predictable in the post-operation period indicative of operation induced ileus.

Pneumopericardium refers to a life-threatening condition that air or gas accumulates in the pericardium. Bricketeau was the first physician who described pneumopericardium in his patient and he proved this diagnosis postmortem in 1844. He also described mill wheel murmur or classical ‘bruit de moulin’ in this condition. Mill wheel murmur is a splashing auscultatory sound when gas in the cardiac chambers exists (1). When intraalveolar pressure rises above atmospheric pressure, alveoli may rupture and air dissects along the bronchovascular sheath and reaches the mediastinum and pericardial cavity. The etiology of pneumopericardium can be classified into four categories: 1- Trauma (both penetrating and blunt) 2-Fistulation between the pericardium and some hollow structures (gastrointestinal tract, the pleural cavity, pulmonary tissue and the bronchial tree) 3- Gas-producing organisms invading the pericardium. 4- Iatrogenic (both diagnostic and therapeutic interventions such as thoracentesis, endo-tracheal intubation, esophagoenterostomy, post-sternal bone marrow aspiration and also major cardiac surgeries) (2). It can be either symptomatic or asymptomatic. The classic symptoms of pneumopericardium include dyspnea and precordial pain. Another clinical sign is shifting tympany in percussion of precordium in recumbent and upright positions.

EKG may show altered ST segments, low voltage due to concurrence of pericarditis or tamponade in such patients. Chest X-ray is useful for confirmation of diagnosis. The presence of air in the pericardium in CXR (double-contoured cardiac silhouette) is the pathognomonic sign of pneumopericardium that is helpful for distinguishing between pneumopericardium and pneumomediastinum (3). Pneumomediastinum is defined as the collection of air or gas in the mediastinal space. Laennec was the first physician who described a case of pneumomediastinum after traumatic injury. The symptoms of pneumomediastinum sometimes are the same as pneumopericardium. Common symptoms of pneumomediastinum include chest pain, dyspnea, fever and dysphonia and throat pain. Other findings in physical examination are subcutaneous air and Hamman sign (precordial systolic crepitation and diminution of heart sounds). Pneumopericardium is less common than pneumomediastinum except after cardiac surgeries. Pneumopericardium and pneumomediastinum may co-exist in some conditions. Tension pneumopericardium is a lethal condition that rarely occurs after cardiac surgery (4). Chest radiography in pneumomediastinum may show radiolucent streaks along the heart margins, around the trachea or retrosternal space, other features of pneumomediastinum in chest radiography are tubular artery sign (lateral chest radiograph shows a radiolucent area around the right pulmonary artery), thymic sail sign (thymic lobes shift upward in infants) and continuous diaphragm sign (when central parts of the diaphragm are visible due the accumulation of air between the diaphragm and the pericardium) (5). Pneumopericardium and pneumomediastinum, although rare, may arise in patients after cardiac surgeries. Physicians should be aware of the possibility of these complications and such patients need to be observed closely due to the possibility of cardiac tamponade or cardiac decompensation.
